# *In Vitro* and *In Vivo* Study of Poly(ethylene glycol) Conjugated Ibuprofen to Extend the Duration of Action

**DOI:** 10.3797/scipharm.0911-07

**Published:** 2011-03-20

**Authors:** Anjali Nayak, Anurekha Jain

**Affiliations:** B. R. Nahata College of Pharmacy, Mandsaur 458001, India

**Keywords:** Ibuprofen, Poly(ethylene glycol), Conjugation, Analgesic, Anti-inflammatory

## Abstract

Ibuprofen–polyethylene glycol (PEG) conjugates (PEG-Ibu) were prepared and their potential as a prolonged release system was investigated. Two PEG-Ibu conjugates were synthesized from Ibuprofen and PEG with two different molecular weights by esterification in the presence of DCC and DMAP. The PEG-Ibu conjugates were characterized by FT-IR, ^1^H NMR, Mass spectroscopy and DSC analysis. The solubility study in aqueous system showed an increase in solubility of conjugates. The dissolution / hydrolysis studies showed a specific acid–base catalysis pattern dependent on the pH of the medium. This indicated a good chemical stability in aqueous buffer solution of acidic medium and the extended release behavior was found in both prodrugs after 9 hour. The results demonstrate that, in the same condition, the rate of hydrolysis for PEG_4000_-Ibu is slower than other. The Writhing induced by acetic acid experiment and paw edema test after oral administration showed that both conjugates had extended analgesic and anti-inflammatory effects compared with Ibuprofen. These results suggest that PEG-Ibu could be a promising NSAID prodrug with an extended pharmacological effect owing to delayed-release of parent drug.

## Introduction

Non-steroidal anti-inflammatory drugs (NSAIDs) are usually poorly soluble in water [1] and they frequently cause gastrointestinal side effects such as gastric ulceration, bleeding and perforation [2, 3]. Ibuprofen is a non-steroidal anti-inflammatory drug with well known anti-inflammatory, antipyretic and analgesic properties. Chemically it is 2-(4-*iso*-butylphenyl)-propanoic acid and is poorly soluble in water [4, 5]. It is most commonly administered orally and is rapidly absorbed to reach its maximal plasma concentration with in 2 hrs [6]. However, it has a short biological half-life of 2 hrs, which means that frequent doses are required to maintain the therapeutic efficacy over extended time periods.

These problems can be solved by the preparation of polymeric prodrug backbones which can contain drugs in a physically bound (dissolved, dispersed, included or adsorbed) state or by true chemical linkages along the polymer backbone or as side groups. These latter systems, in which drugs are delivered by chemically or biologically induced cleavage of the covalent bonds, allow one to achieve a more constant release of the drug for long periods of time. The use of polymers as prodrugs of bioactive agents can thus decrease the required dose, and then the toxicity of the drug, making its solubility and therapeutic efficiency better [7]. In modern era, some NSAIDs such as Ibuprofen, Indomethacin, Ketoprofen and Diclofenac were covalently attached to various polymer backbones and their hydrolysis studied.

The biologically active drug and a polymer conjugation is one of the numerous methods for changing and controlling the pharmacokinetics, biodistribution and often toxicity of these compounds [8–13]. The preparation of a drug-polymer conjugate has been reported widely in many reviews [14–21]. Recently, polymeric prodrugs having Ibuprofen pendent groups were prepared by the free radical polymerization of new polymerizable acrylic monomer with various co monomers. For controlled drug release, this Ibuprofen-dextran ester prodrug improved Ibuprofen solubility and achieving prolonged release properties [20–22]. α-methyl, ethyl and propyl glucopyranoside esters conjugate of Ibuprofen and glyceride prodrugs was reported to have less ulcerogenicity with better anti-inflammatory/analgesic action than their parent drug when administered orally [24, 25]. A conjugate with β-cyclodextrin was prepared by covalent bonding to one of the primary hydroxyl groups of β-cyclodextrin for enhanced Solubility and Dissolution [26, 27]. Drugs that contain reactive functional groups such as carboxyl or hydroxyl groups can be converted to a wide variety of polymerizable derivatives.

In this study poly (ethylene glycol) is being used as the carrier polymer, because it is known to be non-toxic, non-antigenic, non-teratogenic, non-immunogenic, biocompatible, available in variety of molecular weights, linear, uncharged, and amphiphilic polymer, soluble in water and in most organic solvents and has solubilizing properties, rapidly eliminated from the body, and has been approved for a wide range of biomedical applications [15–18, 28]. Considering all these general properties and advantages of PEG, in this research work, we selected PEG as a polymer drug carrier and Ibuprofen as a small molecular drug which was covalently linked to PEG for preparing PEG–Ibu prodrug. By using PEGs with different molecular weights, two polymer prodrugs were synthesized, and their detailed molecular structures were characterized via ^1^H NMR and FTIR techniques. This study aimed to suggest that PEG-Ibu prodrug obtained could be a promising NSAID prodrug with improved solubility, Furthermore, study in vitro on drug release from these prodrugs in various media demonstrated that extended pharmacological effect owing to delayed-release of parent drug for Ibuprofen has been achieved significantly.

## Experimental

### Material

Ibuprofen was obtained from Unisule, sonipat. 4-Dimethylaminopyridine (DMAP) and N,N′-dicyclohexylcarbodiimide (DCC) were obtained from Spectrochem, Mumbai. Poly(ethylene glycol) with a molecular weight of 600 and 4000 was obtained from E.Merck, Mumbai. All other chemicals were of reagent grade and used without further purification.

### Analytical methods

The infrared absorption spectra of the Ibuprofen, PEG and PEG-Ibu were obtained using a FT-IR spectrophotometer (FT/IR- 8400 S, Shimadzu, Japan). The samples were pressed into a potassium bromide pellet before obtaining their IR absorption spectra. The ^1^H NMR spectra were obtained on a Bruker 400 Spectrometer (Bruker Avance-II 400 NMR Spectrometer SAIF, Panjab University, Chandigarh). Ultraviolet–visible spectra were recorded on a Thermospectronic UV 1 Double beam spectrophotometer (Merck, Mumbai). DSC (Shimandzu corporation, DSC-TA-GOWS-(U220 230V), Serial no. C-30454700909) was used to determine the thermal behaviors of Ibuprofen and prodrugs.

## Methods

### Preparation and characterization of the PEG-Ibuprofen conjugate

#### Preparations of the PEG_600_-Ibuprofen conjugate (PEG-Ibu)

Poly (ethylene glycol)*_600_* 5.38 gm. (8mmol) and DCC 6.592 gm. (16mmol) were dissolved in methylene chloride followed by the addition of Ibuprofen 3.3gm (16mmol) and DMAP 0.293gm. (2.4mmol). The mixture was stirred, and a white precipitate of dicyclohexylurea (DCU) was formed after 15–30 min. The reaction was monitored by thin layer chromatography (TLC). After approximately 6–8 h, the precipitate was filtered and the filtrate was evaporated to dryness. The resulting residue was dissolved in acetone, filtered to further remove any DCU, and evaporated again. 10% sodium bicarbonate was used to separate the PEG–Ibu from free Ibuprofen. Column chromatography was used to separate the PEG–Ibu from the reaction mixture. In order to remove the DMAP, a methanol/methylene chloride (9/91): hexane/ethyl acetate (1/2) mixture at a ratio of 20:1 was used as the mobile. The purified products were dried under vacuum. The pure *PEG_600_*–Ibu conjugate with the yield of 60.49% was obtained. The fact that there was no free drug of Ibu existing in polymer prodrug was confirmed by thin layer chromatography and DSC measurements.

#### Preparations of the PEG_4000_-Ibuprofen conjugate (PEG_4000_-Ibu)

The same procedure was used to prepare *PEG_4000_-Ibu* as mentioned above. In this case, Poly (ethylene glycol)_4000_ 8 gm. (2mmol) and DCC 1.648 gm. (4mmol) were dissolved in dichloromethane followed by the addition of 2-(4-isobutylphenyl) propanoic acid (Ibuprofen) 0.825gm. (4mmol) and DMAP 0.1466 gm. (0.6mmol). Yield: 57.57%

### Determination of Drug Solubility

To determine the drug solubility, an excess amount of pure Ibuprofen, *PEG_4000_-Ibu* conjugate and *PEG_600_-Ibu* conjugate were added to distilled water and Phosphate Buffer (pH 7.4). This suspension was stirred at room temperature for 24 hours with a Rota spin test tube Roter. After that the containers (test tubes) were left overnight for saturation. Then these saturated solutions were centrifuged at 10000 rpm the help of macro centrifuge and the supernatant was filtered via Whatman filter paper. The concentration of Ibuprofen was determined spectrophotometrically at 222 nm [29–31].

### Drug release tests via hydrolysis

The hydrolysis rates of the synthesized conjugates were studied in vitro in artificial gastric juice pH 1.2 and in Phosphate buffer solution pH 7.4. The reaction was monitored by UV-Visible spectrophotometer for the increase in concentration of free drug with time.

#### (a) Hydrolysis in 0.2 M hydrochloric acid buffer (pH 1.2)

PEG_600_-Ibu and PEG_4000_-Ibu (wt. equivalent to 10 mg of Ibuprofen) were taken and introduced in 900 ml HCl buffer (pH 1.2) taken in the separate baskets in USP dissolution apparatus type-I and were kept in a constant temperature bath at 37 ± 1 °C. The solutions were occasionally stirred and 5 ml aliquot of both conjugates were withdrawn at various time intervals and were transferred to 10 ml volumetric flasks. The sink conditions were maintained by replacement of equal volume of fresh dissolution medium. Standerd curve were made by pure Ibuprofen in buffer solution using UV spectrophotometer at 222nm. The concentration of Ibuprofen released was analyzed and calculated by comparing the slope of standard curve at 222nm. Experiments was repeated three times.

#### (b) Hydrolysis in 0.2 M phosphate buffer (pH 7.4)

Same procedure as described in (a) was followed for PEG_600_-Ibu and PEG_4000_-Ibu, where instead of HCl buffer, phosphate buffer pH 7.4 was used. Percentage of drug released was calculated. Studies were carried out in triplicate.

### Biological Evaluation

All the animals were obtained from the animal house of the Department of Pharmacology, B.R. Nahata College of Pharmacy, Mandsaur and its animal facility is approved by CPCSCA (Reg. No. 918/ac/05/CPCSEA). The experimental protocols for the same have been approved by Institutional Animal Ethics Committee. The analgesic activity was carried out on Swiss albino mice by the Siegmund et al. method; anti-inflammatory activity of the compounds was carried out on Wistar rats by the Winter et al. method [25, 32].

### Writhing Induced by Acetic Acid

Analgesic activity was carried out by using acetic acid induced writhing method in Swiss albino mice (25–30 g) of either sex. A 1% v/v solution of acetic acid was used to induce writhing. Test compounds were administered orally 3 h prior to acetic acid injection. Number of writhing for 10 min in control and test compounds were counted and compared. To evaluate the analgesic activity of the conjugates 4 groups of animals (n = 6) were examined. A first group of rats was used as a control receiving vehicle 5 ml kg^−1^, while group II received Ibuprofen (20 mg kg^−1^), groups III and IV received PEG_600_-Ibu conjugate and PEG_4000_-Ibu conjugate (52.60 mg kg^−1^ and 214 mg kg^−1^ respectively), where the dose of conjugate was molecularly equivalent to Ibuprofen. A stock solution of 0.2, 0.52 and 2.14 mg ml^−1^ was prepared as a homogeneous suspension in aqueous solution of sodium CMC (0.5% w/v) and each animal received 2.0–2.2 ml of the respective drugs orally. Acetic acid was administered intraperitoneally 1 ml/100 g body weight of the animal. Analgesic activity was measured as percent decrease in writhing in comparison to control. The percent Inhibition was calculated using the following equation:

$$\%\;\text{Inhibition}\;=\;100\;-\;\frac{\text{number}\;\text{of}\;\text{writhings}\;\text{in}\;\text{test}}{\text{number}\;\text{of}\;\text{writhings}\;\text{in}\;\text{control}}\times 100$$

### Carrageenan-Induced paw edema

The anti-inflammatory activity was evaluated using carrageenan-induced paw edema on rat method. To evaluate the anti-inflammatory activity of the conjugates 4 groups (n = 6) of Wistar rats (150–200 g) were examined. A first group of rats was used as a control without using the drug, group II received Ibuprofen 20 mg kg^−1^, received PEG_600_-Ibu conjugate and PEG_4000_-Ibu conjugate (52.60 mg kg^−1^ and 214 mg kg^−1^ respectively), where the dose was molecularly equivalent to the Ibuprofen. A stock solution of 0.2, 0.52 and 2.14 mg ml^−1^ was prepared as a homogeneous suspension in aqueous solution of sodium CMC (0.5% w/v) and each animal received 2.0– 2.2 ml of the respective drugs orally. Thirty minutes after administration of drugs, each rat received a subplanter injection of 0.1 ml of 1% carrageenan solution in its left hind paw. The swelling volume of the paw was measured before (time 0) and at 0.5, 1, 2, 3, 4, 24 h after the carrageenan injection. The measurement of the hind paw volume was carried out using an Ugo Basile Plethysmometer before any treatment (*V*o) and in any interval (*V*t) after the administration of the drugs. The percentage increase in the paw volume was calculated from the normal paw volume. The percentage of swelling inhibition was calculated using:

$$\text{Inhibition}\;(\%)\;=\;\frac{{\left(\text{Vt}\;-\;\text{Vo}\right)}_{\text{control}}\;-\;{\left(\text{Vt}\;-\;\text{Vo}\right)}_{\text{treated}}}{\left(\text{Vt}\;-\;\text{Vo}\right)}\times 100$$

(*V*t and *V*o relates to the average volume in the hind paw of the rats (*n* = 6) before any treatment and after anti-inflammatory agent treatment, respectively.

### Statistical Analysis

All the results are expressed as mean ± S.E.M. Statistical evaluation was performed using analysis of variance followed by Dunnet’s *t*-test for sub group comparison. A *P* value <0.001 was considered significant.

## Results and discussion

### Preparation of PEG-Ibu conjugate

**Fig. 1.**
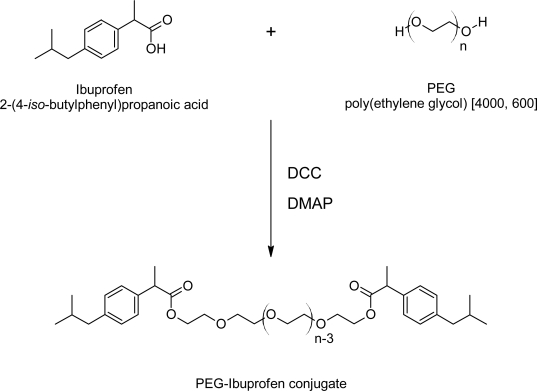
Synthesis of the PEG-Ibuprofen conjugate

In this work, *PEG-Ibu* was synthesized by the esterification of the carboxyl group of Ibuprofen with the hydroxyl group of PEG at room temperature, as shown in Fig. 1.

**Fig. 2.**
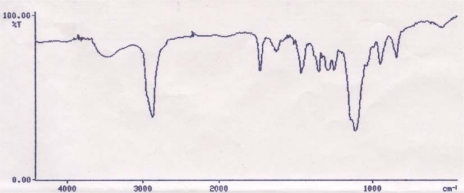
FTIR OF PEG_4000_-Ibu conjugate

The esterification was performed using DCC as the coupling agent and DMAP as a catalyst [33]. The conjugation between Ibuprofen and PEG was confirmed by FT-IR and ^1^H NMR. The position of peak on IR spectrum for PEG_4000_-Ibu conjugate was assigned as follows; IR (pellet): 2876.8, 1731, 1512, 1113.cm^−1^. When conjugated, the hydroxyl groups of Ibuprofen and PEG are expected to disappear due to the esterification process. The carbonyl peak of Ibuprofen appearing at 1710 cm^−1^ due to intermolecular hydrogen bonding will be shifted to a higher frequency after conjugation with PEG. In addition, larger and sharper aliphatic stretching band of C-H will appear, due to the increased number of C-H bonds by PEG. As shown in Fig. 2, there was no hydroxyl peak in the PEG_4000_ spectrum and the carbonyl peak shifted to 1731 cm^−1^. Also, the larger and sharper C-H stretching band appeared at 2876.8 cm^−1^.

**Fig. 3.**
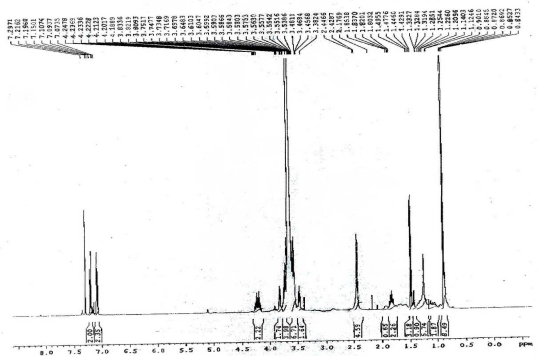
^1^HNMR spectra of PEG 4000–Ibuprofen Conjugate

At room temperature, PEG_600_-Ibu was oily, and the PEG_4000_-Ibu was solid. All the conjugates were soluble in water and organic solvents such as ethanol, chloroform and dichloromethane. Fig. 3 shows the chemical shift of the PEG_4000_-Ibu measured by ^1^H NMR. PEG_4000_-Ibu: ^1^H NMR (400 MHz, CDCl_3_, ppm): δ 0.90 [d, 12H,-CH(CH_3_)_2_], 1.47 [d, 6H, -CHCH_3_], 1.80–1.87 [m,2H, -CH(CH_3_)_2_], 2.42 [d, 2H, -CH_2_], 3.38–3.83 [m, -O(OCH_2_CH_2_)n], 3.69–3.75 [q, 2H, -CHCH_3_], 4.17–4.24 [m, 4H, CO_2_CH_2_], 7.07–7.29 [m, 8H, ArH]. The pegylation was confirmed by disappearance of the signal of the carboxylic acid at 11.23 ppm and appearance of -CO_2_CH_2_-protons at 4.2 ppm.

The position of each peak in IR and NMR spectrum for PEG_600_-Ibu: IR (KBr pellet): 2885.8, 1734.2, 1467.2, 1114.1 cm^−1^. ^1^H NMR (400 MHz, CDCl_3_, ppm): δ 0.89 [d, 12H, -CH(CH_3_)_2_], 1.46 [d, 6H, -CHCH_3_], 1.53–1.58 [m, 2H, -CH(CH_3_)_2_], 2.44 [d, 2H, -CH_2_], 3.54–3.72 [m, -O(OCH_2_CH_2_)n], 3.65–3.72 [q, 2H, -CHCH_3_], 4.10–4.19 [m, 4H, CO_2_CH_2_], 7.06–7.21 [m, 8H, ArH].

**Fig. 4.**
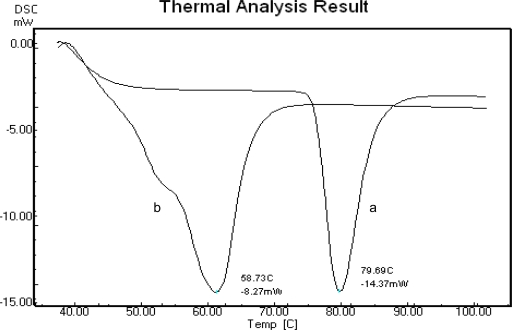
DSC-thermogram of pure Ibuprofen (a), *PEG_4000_-Ibu* conjugate (b)

Additionally, thin layer chromatography, DSC was used for determining free Ibuprofen in polymer prodrug, and the results as shown in Fig. 4. Pure Ibuprofen melting point was at 79.69°C on its DSC thermogram, and *PEG_4000_-Ibu* was at 58.73°C. However no peak was visible near the Ibuprofen’s melting point for the polymeric conjugate thermogram.

### Aqueous Solubility

**Fig. 5.**
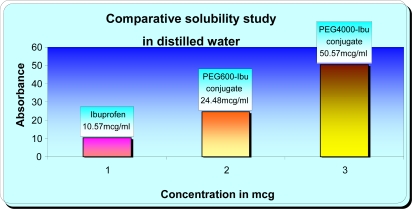
Comparative Solubility Study in Distilled Water

**Fig. 6.**
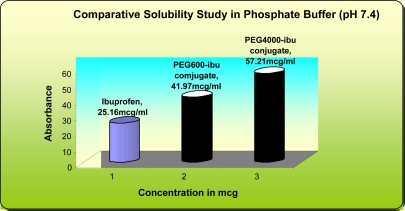
Comparative Solubility in Phosphate Buffer (pH 7.4)

Physicochemical parameter such as aqueous solubility has been shown to influence membrane flux, therapeutic activity, and pharmacokinetic profiles of medicines. The solubility of Ibuprofen in distilled water has been previously found to be 10.57 mcg/mL. In this study, the solubility of PEG_600_-Ibuprofen and PEG_4000_-Ibuprofen conjugates in distilled water were 24.48 mcg/mL and 50.57 mcg/mL respectively. The solubility of Ibuprofen in phosphate buffer (pH 7.4) has been previously found to be 25.16 mcg/mL and solubility of PEG_600_-Ibuprofen and PEG_4000_-Ibuprofen conjugates in phosphate buffer (pH 7.4) were 41.97 mcg/mL and 57.21 mcg/mL respectively. As may be noted in Fig. 5 and 6, Ibuprofen water solubility was less while for PEG_600_-Ibuprofen and PEG_4000_-Ibuprofen conjugates an increase of this parameter was observed. From the solubility data we can conclude that PEG_4000_-Ibu conjugate is more hydrophilic in nature.

### In vitro Hydrolysis Studies

In order to obtain some preliminary information about the potential use of the PEG-Ibu as a drug delivery system for a prolonged release, hydrolysis process for two prodrugs is studied in buffer solutions at pH 1.2 and 7.4. This study shows whether the prodrugs hydrolyze in these buffer solutions and to what extent, providing an idea of the fate of the prodrug in-vivo.

**Fig. 7.**
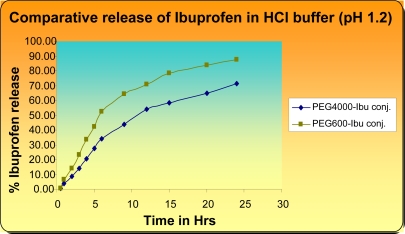
Comparative release of Ibuprofen in HCl buffer (pH 1.2)

On the one hand comparison of the release profile of Ibuprofen conjugates (PEG_600_-Ibu and PEG_4000_-Ibu conjugates) in 0.2 M HCl buffer (pH 1.2) revealed that PEG_600_-Ibu and PEG_4000_-Ibu conjugates were hydrolysed to an extent of 64.35% and 43.9% at pH 1.2 after 8 hrs and this percentage still increases by time reaching 87.26% and 71.2% after 24 hrs for PEG_600_-Ibu and PEG_4000_-Ibu conjugate respectively (Fig. 7).

**Fig. 8.**
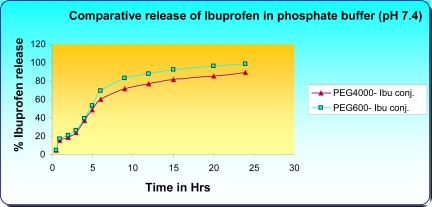
Comparative Release of Ibuprofen in Phosphate Buffer (pH 7.4)

On the other hand, Ibu-release from the conjugates in 0.2 M phosphate buffer (pH 7.4) amounted to 83.1% (PEG_600_-Ibu) and 71.2% (PEG_4000_-Ibu) after 8 hrs and further increased 98.3 and 89.0% in 24 hrs for PEG_600_-Ibu and PEG_4000_-Ibu conjugate respectively (Fig. 8). The hydrolysis rate of Ibuprofen from PEG_600_-Ibu and PEG_4000_-Ibu conjugates was calculated to be 8% and 5% per hour at pH 1.2 and 16% and 9% at pH 7.4, respectively. This study shows that hydrolysis rate of Ibuprofen from PEG_4000_-Ibu is slower than other.

The dissolution / hydrolysis studies of ester prodrugs indicated a better chemical stability in aqueous buffer solution of acidic pH 1.2 than at nearly neutral pH 7.4. In case of oral administration, this study implies that about 88–90 % intact conjugate reach the intestine provided a mean gastric residence time of the conjugates for two hours.

Another aspect was also cleared that hydrolysis of conjugates decreased with increase in length of PEG and the extended release behavior was found in both conjugates in both pH 1.2 and 7.4 buffer solutions after 8 hrs. to 24 hrs. (Figs. 7 and 8).

### Analgesic Activity

**Fig. 9.**
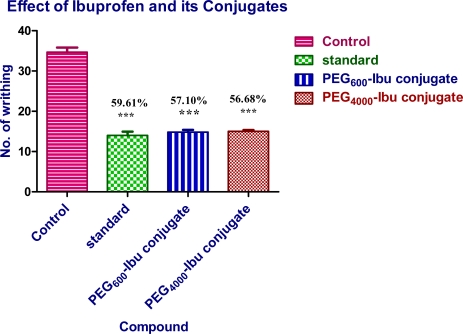
Effect of ibuprofen and its conjugates (PEG_600_-Ibu and PEG_4000_-Ibu) on acetic acid induced writhing in mice. Data represent mean values (±S.D.) and percent inhibition (%) compared to the control animals. Statistical differences versus control group are indicated by asterisks {N = 6, one-way ANOVA, (p< 0.05 (*), p< 0.01 (**) and p< 0.001 (***)}.

Analgesic activity was carried out by using acetic acid induced writhing method in Swiss albino mice (25–30 g) of either sex. A 1% v/v solution of acetic acid was used to induce writhing. Test compounds were administered orally 3 h prior to acetic acid injection. The PEG_600_-Ibu conjugate and PEG_4000_-Ibu conjugate showed analgesic activity comparable to the parent drug. The percent protection in mice brought about by administration of the drugs. In writhing model, Ibuprofen and all its prodrugs (PEG_600_-Ibu conjugate and PEG_4000_-Ibu conjugate) presented remarkable reduction of writhing number if compared to control group (Fig. 9).

Ibuprofen administrated 3 hr. prior to testing produced a significant decrease (59.61%) in writhing number versus control values Moreover, both conjugate PEG_600_-Ibu conjugate and PEG_4000_-Ibu conjugate were equally effective in reducing the writhing response (57.10% and 59.02% respectively) at molecular equivalent quantity. however, no appreciable difference could be observed among all prodrugs with respect to their analgesic activity.

### Anti-inflammatory Activity

Table 2 shows the effect of Ibuprofen, PEG_600_-Ibu and PEG_4000_-Ibu conjugates on the volume of acute inflammatory paw edema in rats caused by carrageenan after oral administration. Although the peak anti-inflammatory effects of PEG_600_-Ibu conjugate and PEG_4000_-Ibu conjugate occurred later than Ibuprofen, but it exhibited a significantly extended anti-inflammatory activity compared with Ibuprofen. The maximum anti-inflammatory activity of prodrugs (PEG_600_-Ibu conjugate and PEG_4000_-Ibu conjugate) was observed at 3–4 h and remained practically constant up to 8 h. The anti-inflammatory activity of free Ibuprofen however decreased with time.

Statistical significance testing using one way analysis of variance showed that the anti-inflammatory activity of ibuprofen and prodrugs were effective in comparison with the control group. However, differences in the potency of anti-inflammatory activity of the prodrugs compared to the free ibuprofen were observed over a long period (8 h). Thus PEG_600_-Ibu conjugate and PEG_4000_-Ibu conjugate were investigated to be a suitable promoiety for Ibuprofen.

## Figures and Tables

**Tab. 2.** Percentage of Inhibition Caused by Ibuprofen and Its Conjugates in Carrageenan-Induced edema in Wistar rats (150–200 g)

**Treatment**	**Dose (mg/kg)**	**1 hr.**	**2 hr**	**3 hr**	**4 hr**	**8 hr**	**24 hr**
Control	–	0.59 ± 0.005	0.68 ± 0.007	0.48 ± 0.009	0.43 ± 0.011	0.32 ± 0.005	0.27 ± 0.006
Standard	20	0.33 ± 0.013**	0.48 ± 0.017**	0.38 ± 0.013**	0.24 ± 0.009**	0.15 ± 0.010^ns^	0.03 ± 0.013^ns^
PEG_600_-Ibu Conjugate	52.60	0.22 ± 0.008***	0.33 ± 0.007***	0.43 ± 0.012**	0.37 ± 0.014**	0.30 ± 0.009**	0.17 ± 0.010**
PEG_4000_-Ibu Conjugate	214	0.25 ± 0.011**	0.36 ± 0.010^**^	0.45 ± 0.018**	0.40 ± 0.014**	0.34 ± 0.007**	0.20 ± 0.011**

Value are expressed in mean ± SEM;

* p< 0.05,

** p< 0.01, and

*** p< 0.001; One-way Anova followed by Dunnet’s test.
